# Differences in Soil Properties and Bacterial Communities between the Rhizosphere and Bulk Soil and among Different Production Areas of the Medicinal Plant *Fritillaria thunbergii*

**DOI:** 10.3390/ijms12063770

**Published:** 2011-06-09

**Authors:** Ji-Yan Shi, Xiao-Feng Yuan, Hui-Rong Lin, Yuan-Qiang Yang, Zong-Yuan Li

**Affiliations:** 1 Department of Environmental Engineering, Zhejiang University, Hangzhou 310029, China; E-Mails: shijiyan@zju.edu.cn (J.-Y.S.); yangyuanqiang86@tom.com (Y.-Q.Y.); 2 College of Life Science, Zhejiang Chinese Medical University, Hangzhou 310053, China; E-Mail: tongxianglizongyuan@hotmail.com (Z.-Y.L.); 3 Key Laboratory of Non-Point Sources Pollution Control, The Ministry of Agriculture of the People’s Republic of China, Hangzhou 310000, China; 4 Department of Environmental Science and Engineering, Xiamen University Tan Kah Kee College, Zhangzhou 363105, China; E-Mail: linhuirong@yahoo.com.cn

**Keywords:** *Fritillaria thunbergii*, geoherbs, bacteria, bulk soil, rhizosphere soil, PCR-DGGE

## Abstract

To explore rhizosphere effects, geographical differences and their effects on the bacterial community associated with the geoherb *Fritillaria thunbergii*, some physicochemical properties of soil samples (3 sampling sites × 2 habitats (rhizosphere and bulk soil)) were measured and the soil bacterial community detected by PCR-denaturing gradient gel electrophoresis (DGGE). Among the three regions, soil pH varied between 4.48 and 7.73 indicating that *F. thunbergii* could grow both in acid and slightly alkaline soil. As the authentic Dao-di producing area, Ningbo showed the highest soil quality with the highest content of organic matter (OM) (2.46%), phosphatase (268 mg kg^−1^ 24 h^−1^) and urease activity (1481 mg kg^−1^ 24 h^−1^). In comparison with the bulk soil, pH, organic carbon content, and phosphatase and urease activities were all lower in the rhizosphere, suggesting that the roots may secrete some unique metabolites in root exudates. Statistical analyses showed that soil properties of Ningbo and Panan in Zhejiang province were more similar to each other than those in Nantong in Jiangsu province. In addition, PCR-DGGE analysis showed that main bacterial population identified in *F. thunbergii* was proteobacteria (18 bands, 55%), acidobacteria (4, 12%), actinobacteria (4, 12%) and bacterioidetes (6, 18%). Overall, soil properties and microbial communities varied not only between the rhizosphere and bulk soil but also among the three regions. We suggest that the plant, together with the soil properties, cooperatively shape the structure of the rhizosphere bacteria, and that the soil properties have a close relationship with the geoherbalism of *F. thunbergii*.

## 1. Introduction

There are many geoherbs (Dao-di Chinese medicinal plants) in China which are endemic to particular areas. Some of these areas have a high reputation for producing plants of authentic medical quality. Geoherbalism is the expression of particular properties of geoherbs, primarily embodied in their quality (including appearance and the content of secondary metabolic products), hereditary and habitat features [1]. *Fritillaria thunbergii* (Family: Liliaceae) is a geoherb with high medicinal value. It is only found in the east of China including in Zhejiang, Jiangsu, Jiangxi and Anhui provinces but only the Ningbo region in Zhejiang province is regarded as an authentic Dao-di producing area [2]. Geoherbalism has a close relationship with the ecological environment, especially with the micro-ecology of the rhizosphere formed by the interaction between plant, soil, rhizosphere organisms and the environment [3]. However, current geoherb research has mainly focused on medicinal properties and investigations of the effects of environmental factors such as soil, water, temperature on primary and secondary metabolites, but reasons for the observed effects have seldom been reported [4]. Wang *et al*. [5] evaluated the producing areas of *F. thunbergii* using a geographic information system, but few studies have investigated in detail the relationship between soil microbes and the geoherbalism of *F. thunbergii.*

The rhizosphere is an area of intensive interaction between plant roots and soil. It is of central importance, not only for plant nutrition, health and quality, but also for microorganism-driven carbon sequestration, ecosystem functioning and nutrient cycling in terrestrial ecosystems [6]. The rhizosphere effect is mainly based on the reproduction and distribution of microorganisms influenced by root growth and the environment [7], and the well-studied rhizosphere effect describes the phenomenon that, in comparison with bulk soil, the biomass and activity of microorganisms is enhanced as a result of exudation of compounds by the root [8]. Karthikeyan *et al*. [9] also found that, in four medicinal plants, the microbial population is greater in the rhizosphere when compared to bulk soil. However, Berg and Smalla [6] observed that some other medicinal plants, for example camomile, thyme and eucalyptus, contained unique antimicrobial metabolites in their exudates which influenced the structure and function of microbial communities. Whether the rhizosphere effect is different for medicinal plants such as *F. thunbergii* needs further research.

With the modernization of traditional Chinese medicine (TCM), its safety has become of growing importance [10]. Toxic heavy metal pollution is one of the main problems. Hence, the content of some heavy metals besides some common elements are measured by inductively coupled plasma-optical emission spectroscopy (ICP-OES). Furthermore, pH, organic carbon content, and enzyme activities in soil are also investigated to explore the rhizosphere effects of *F. thunbergii*. To our knowledge, it is the first study comparing the composition of the bacterial community in the rhizosphere and bulk soil of a medicinal plant by PCR-denaturing gradient gel electrophoresis (DGGE).

## 2. Results

### 2.1. pH, OM and the Activities of Urease and Phosphatase in Soil

**Figure 1:**
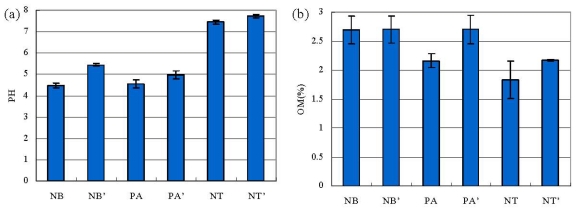

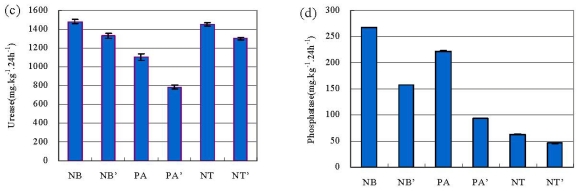
The pH, the content of organic matter and the activities of urease and phosphatase in soil samples of *F. thunbergii*: (**a**) pH; (**b**) organic matter content; (**c**) urease; (**d**) phosphatase. For convenience, the soil samples were named by the abbreviation of the producing area, including PA (the rhizosphere soil in Panan); PA′ (the bulk soil in Panan); NB (the rhizosphere soil in Ningbo), NB′ (the bulk soil in Ningbo); NT (the rhizosphere soil in Nantong) and NT′ (the bulk soil in Nantong).

Among the three regions (Figure 1a), the soil pH was 4.48 in Ningbo and 4.57 in Panan, while it was 7.45 in Nantong, indicating that *F. thunbergii* could grow in acid and slightly alkaline soil. The content of organic matter (OM) and the phosphatase activity both were the highest in the rhizosphere soil in Ningbo region, followed by Panan, and lowest in Nantong region. The urease activity was also highest in the rhizosphere soil of Ningbo region, followed by Nantong region and Panan region (Figure 1b, 1c and 1d). It could be seen that soils in the Dao-di producing area Ningbo region showed the highest OM content and enzyme activities, which suggested that the enzyme activities have a close relationship with the fertility of the soil and soil properties may have a close relationship with the geoherbalism of *F. thunbergii.*

In comparison with the bulk soil, pH is lower in the rhizosphere in each region. The OM content of the bulk soil varied between 2.13% and 2.49%, higher than in the rhizosphere where OM contents ranged from 1.85% to 2.46%. Furthermore, lower activities of both enzymes were observed in the rhizosphere, which is contrary to results in many researches [11], which reported the enzyme activities were higher in rhizosphere than in the bulk soil of *Cunninghamia lanceolata*, *Pinus sylvestris* var. *mongolica* and C*amellia oleifera*.

### 2.2. Element Concentration in Soil

Some research indicated that the medicinal properties of the same medicinal plant from different production areas showed significant differences, mainly due to varying element contents [12]. Among the three regions, elements Al, B, Ca, K, Mg, S, Na, P, Fe, Mn were abundant in soil of *F. thunbergii*, present at grams per kilogram level. Most of them are macro- or micro-nutrients of plants and the order of contents was K > Na > Fe > Ca > B > P > Mn > Mg > S (Table 1). For most elements, the law of content of each element in the three regions is significantly different. Soil in the Dao-di producing area of Ningbo showed the highest concentrations of P, K, Al, Na, S, Zn and Cu and the lowest B content. Moreover, it is interesting that the concentration of some elements such as B, Ca, Fe, Mg and Mn were higher in Nantong than in Ningbo and Panan, and those of Al, K, Na were lower. In Ningbo, the highest concentrations of P and K are also found, probably reflecting accelerated microorganism decomposition and release of organic and non-organic P and K [13]. Overall, the soil properties of Ningbo and Panan in Zhejiang province were relatively more similar to each other than to those of Nantong in Jiangsu province. According to the “National Soil Environmental Quality Standard” (GB15618-1995), soil concentrations of the heavy metals Cr, Cu, Pb, Zn and Cd should be less than 150, 150, 250, 200 and 0.3 mg kg^−1^, respectively. It can be seen from Table1 that the contents of all heavy metals except for Cd were well below the national standards, and the Cd content ranged from 0.51–6 mg kg^−1^, exceeding the national standard. Futhermore, in comparison with the bulk soil, higher concentrations of Al, B, Ca, P and S were observed in the rhizosphere, but no obvious pattern for most elements was observed.

Statistical analyses, including Pearson correlation analysis and principal component analysis (PCA), were done to further explore relationships and investigate the major cause of spatial variability. Pearson correlation analysis showed that a significant positive correlation between soil pH and urease, Ca, Fe, Mg, Mn, B, Co, Cr, Cu and Zn concentrations, whereas a significant negative correlation was found between soil pH and phosphatase and element Al, Na and Cd, which indicated pH was an important factor in soil. In fact the effect of pH changes on the microbial activity is the main reason for the changes on enzyme activities in the soil [14]. Phosphatase showed a significant negative correlation with many elements such as Ca, Fe, Mg, Mn, B, Co and Cr but a positive correlation with OM, Al, Na, K, P, Cd and Pb. Meanwhile, urease showed a significant negative correlation with Al concentrations and a positive correlation with Ca, Mg, P, S, B, Co, Zn, Cu Cr and Pb (Table 2), which showed that the activities of enzymes in the soil had a close relationship with many heavy metals. Moreover, many elements showed significant correlation with each other, indicating that each element in soil was not isolated but influenced each other. Nevertheless, Shannon index showed, statistically, non-significant correlations with other soil properties, suggesting that bacteria in the soil had no correlation with enzyme activities, which were in accordance with others’ results [15,16].

**Figure 2:**
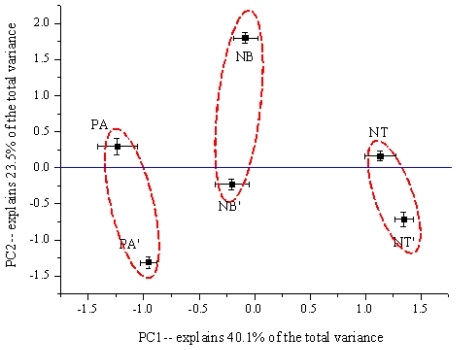
Principal component analysis (PCA) of all soil samples of *F. thunbergii*. X axis was defined by PC1 which represented 40.1% of the total variance and Y axis was defined by PC2 which represented 23.5% of the total variance. Abbreviations: NT: The rhizosphere soil in Nantong; NT′: The bulk soil in Nantong; PA: The rhizosphere soil in Panan; PA′: The bulk soil in Panan; NB: The rhizosphere soil in Ningbo, NB′: The bulk soil in Ningbo.

PCA results showed that the first four principal components (PC1-4), with eigenvalues >1, explained 91.0% of the total variability among the 22 variables in the original data, where PC1, PC2, PC3, and PC4 contributed 40.1%, 23.5%, 17.7%, and 9.7% of the total variance, respectively, showing that a four-factor model could explain 91.0% of the test data. The pH and urease activity as well as Cr, Mg, Co, Ca, Cu, and Zn concentrations showed higher weights in PC1; Al, Cd and Pb concentrations loaded highly in PC2. Since clustering and trends around PC 1-2 represented 63.6% of the total variance, a two-dimensional scores plot was performed using Origin 8.0. From the X axis defined by PC1, the scores for each of the two samples from the same producing area were very similar, and then it was obviously divided into three groups (Figure 2). The rhizosphere and bulk soil were easily distinguished from the Y axis, defined by PC2, since the scores of the rhizosphere soil of *F. thunbergii* were all more than zero, whereas those in the bulk soil were well below zero. Soil properties from the Panan and Ningbo regions were more similar to each other than those from Nantong.

### 2.3. The Structure of Bacterial Community in Soil

**Figure 3:**
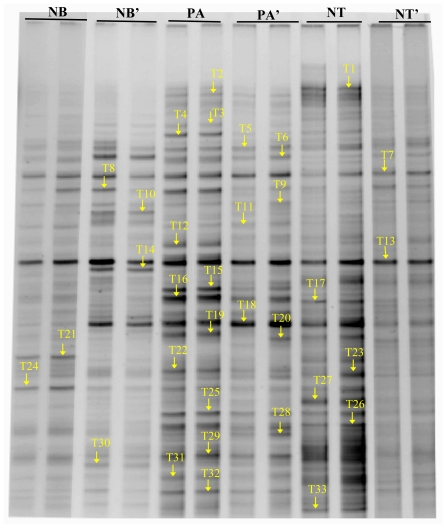
PCR-DGGE profiles of soil bacteria in different producing areas. (T1-T33: 33 bands recovered for sequencing). Only two replicates in each sample were selected in this experiment since DGGE profiles of triplicate samples were highly reproducible by the optimization of DGGE running conditions. Abbreviations: NT: The rhizosphere soil in Nantong; NT′: The bulk soil in Nantong; PA: The rhizosphere soil in Panan; PA′: The bulk soil in Panan; NB: The rhizosphere soil in Ningbo, NB′: The bulk soil in Ningbo.

**Figure 4:**
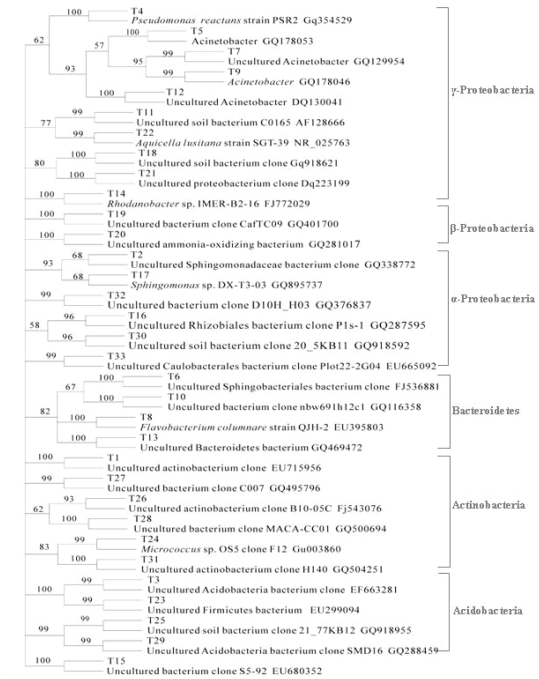
Phylogenetic tree of 16S rDNA clones obtained from samples of *F. thunbergii* growing soils.

To obtain further information about the dominating bacterial populations of *F. thunbergii*, we chose thirty-three prominent bands but without isolating a certain band of every soil sample for cloning and sequencing (Figure 3). The similar band at the same location in the DGGE fingerprint in all soil samples was attributed to the same microorganism. The closest relative strains available in the GenBank database were obtained using the Blast Program (Figure 4). The similarity between 33 OTUs (operation taxonomic unit) and the closest sequences were all more than 94%, indicating that they probably belong to the same genus. It was found from sequencing results that the 33 bands belonged to 23 genus, 18 families and 4 phyla including proteobacteria (18 bands, 55%), acidobacteria (4, 12%), actinobacteria (4, 12%) and bacterioidetes (6, 18%). Clones from proteobacteria were the most diverse and abundant, and included the classes alpha-proteobacteria, beta-proteobacteria, and gamma-proteobacteria, suggesting they played an important role in the growth of *F. thunbergii*.

A comparison of the DGGE patterns among the three regions revealed obvious differences in the composition of the bacterial community between the rhizosphere and bulk soil. There were not only some specific bands in each region but also some bands in common, just as shown in Table 3. Among them, band T7, T8, T13, T18 and T32 with various intensities were found simultaneously in all soil samples, indicating that Uncultured *Acinetobacter* sp. clone GI8-sp-H16, *Flavobacterium columnare* strain QJH-2, Uncultured Bacteroidetes bacterium, Uncultured soil bacterium clone 205KE05 and Uncultured bacterium clone D10HH03 were the predominant bacteria in soil of *F. thunbergii*. On the other hand, bands T21 in the Ningbo, bands T16 and T19 in Panan, and band T27, T33 in Nantong were present in each region mainly due to the effect of environmental factors, such as soil properties, *etc.*

Comparison of the rhizosphere and bulk soil from the same producing area also showed obvious differences in the number and intensity of bands, suggesting the growth of the plant would affect the structure of the rhizo-bacteria community. In particular, Uncultured Sphingobacteriales bacterium (bands T6), Uncultured proteobacterium clone (T21) and Uncultured bacterium clone MACA-CC01 (T28) only appeared in rhizosphere whereas *Acinetobacter* sp. 71A1 (T5), Uncultured bacterium clone nbw691h12c1 (T10), *Rhodanobacter* sp. IMER-B2-16 (T14) and Uncultured soil bacterium clone 20_5KB11 (T30) only in the bulk soil in Ningbo region. In Panan region, Uncultured actinobacterium clone (T1), Uncultured *Acinetobacter* sp. clone JEL30 (T12), Uncultured Rhizobiales bacterium clone P1s-1 (T16) and Uncultured bacterium clone CafTC09 (T19) were found only in the rhizosphere soil and Uncultured ammonia-oxidizing bacterium isolate (T20) only in the bulk soil. Meanwhile, there were many bands such as Uncultured Sphingomonadaceae bacterium clone (T2), *Pseudomonas reactans* strain PSR2 (T4), Uncultured proteobacterium clone (T21), *Micrococcus* sp. OS5 clone F12 (T24), Uncultured actinobacterium clone B10-05C (T26), Uncultured bacterium clone C007 (T27) and Uncultured Caulobacterales bacterium clone (T33) in the rhizosphere but band *Flavobacterium columnare* strain QJH-2 (T8) only in bulk soil in Nantong. On the whole, PCR-DGGE analysis showed clear differences in the bacterial community composition of non-rhizosphere soil and the fritillaria rhizospheres, In addition, the species diversity based on Shannon index was higher in the rhizosphere soil than in non-rhizosphere soil [17], which indicated the root exudates secreted by *F. thunbergii* would promote the accumulation of some bacteria, at the same time restrain some other bacteria, leading to higher bacterial diversity in the rhizosphere soil.

## 3. Discussion

In contrast to what we know about the biodiversity of microorganisms, microbial biogeography is controlled primarily by edaphic variables, especially by pH [18], which has complicated effects on soil microbial communities by influencing the availability of nutrients, microbial adsorption, and production and secretion of extra cellular enzymes, as well as the growth of microorganisms. This study showed that *F. thunbergii* can adapt to a broad pH range from acidic to alkaline. Liu *et al*. [19] thought the alkaline pH of soil may be the reason for the high total nutrient level of *F. thunbergii* in Nantong. Our research also found that rhizo-bacteria diversity and the content of alkaloid of *F. thunbergii* in Nantong were both the highest among the producing areas [17]. Although the location of the three producing areas of *F. thunbergii* varied, the climate conditions were similar in these areas and would not cause the difference of the microbial composition in the soil. Hence, there was no doubt that soil properties such as pH may play an important role in the quality of *F. thunbergii* and the soil of alkaline pH may be more appropriate for the growth of *F. thunbergii.*

Due to their high sensitivity and rapid response, soil biological properties including microbial enzymatic activity are appropriate indicators of soil quality under different agricultural systems [20]. In general, overall enzymes in soil derive from the activities of microorganisms, root exudates and the decomposition of plants and animals [21,22]. Up to now, most research showed that enzyme activities were higher in the rhizosphere than in the bulk soil [11,23], yet the contrary was found in this study where the activities of phosphatase and urease were both higher in the bulk soil. Since it is a medical material for treating upper respiratory tract infection and the active components are certain alkaloids, *F. thunbergii* could provide some unique anti-enzyme activity metabolites in the exudates which would affect the structure of rhizo-bacteria community. In fact, it was estimated that the existing huge diversity of plant species ranged from 310,000 to 422,000 species [24] and corresponding secondary metabolites of plants [25] affected below-ground diversity [6]. In this study, there were some bacteria which only appeared, and some bacteria which disappeared, in the rhizosphere soil, compared to the bulk soil, which indicated that the root exudates may affect the structure and function of microbial communities and then influence the enzyme activities. Briefly, as a medicinal plant, the rhizosphere effect of *F. thunbergii* is slightly different and the active components in potential exudates from *F. thunbergii* need further research.

The result of PCR-DGGE showed that the most abundant clones extracted from the rhizosphere samples were identified as proteobacteria, acidobacteria, actinobacteria and bacterioidetes, suggesting that the soil in which *F. thunbergii* grows suits the growth of these kinds of bacteria. From this group, bacterial species were identified that are known to have disease prevention or plant growth promoting properties such as *Pseudomonas* (band T4), *Flavobacterium* (band T8) and *Bacillus* (band T13) [26]. Moreover, as shown in Figures 3 and 4, the structures of rhizo-bacteria community were different not only among different areas but also between rhizosphere and bulk soil, suggesting that both factors, soil properties as well as plant species, influenced the structure and function of microbial communities.

## 4. Experimental Section

### 4.1. Soil Sample Collection

**Figure 5:**
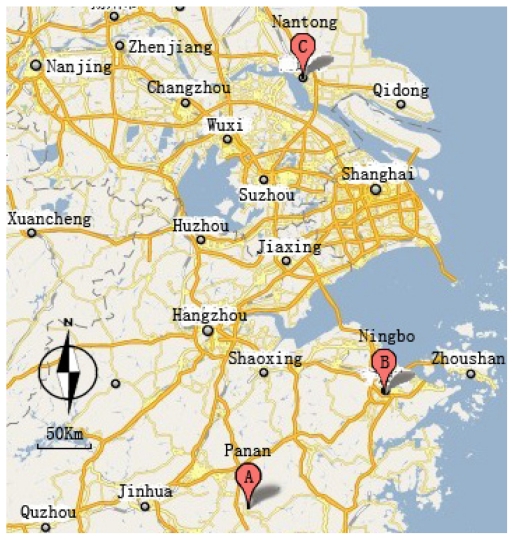
Three sampling locations of *F. thunbergii*.

The most representative of three producing areas of *F. thunbergii* were chosen for the research: the Nantong, Ningbo and Panan regions as the Ningbo region in Zhejiang province is an authentic Dao-di producing area; the Panan region in Zhejiang province as a non-authentic Dao-di producing area; and the Nantong region in Jiangsu province also as a non-authentic Dao-di producing area but in a different province. The main climate conditions in these regions are as follows: the annual temperature ranged from −7 to 38 °C; the annual rainfall is 1300–1450 mm; the altitude is 300–700 m and yellow soil. Soil samples were taken in April 2009 from the rhizosphere and bulk soil of three regions, the locations of which are shown in Figure 5. At least fifteen plants of *F. thunbergii*were randomly selected from five spots in each sampling site and the roots were shaken gently to separate soil not tightly adhering to the roots for each replica. The soil in 10–15 cm depth that remained attached to the roots was considered as rhizosphere soil and that the soil in 10–15 cm depth in which *F. thunbergii* had no plants growing nearby, was bulk soil [27]. Each sample was taken in triplicate. Part of the samples were kept moist in the dark at −20 °C for microbial diversity analysis and at 4 °C to assess soil enzyme activity. The remaining soil was air-dried at ambient temperature, crushed and sieved through a 0.2 mm mesh for analysis of pH, organic matter and element contents.

### 4.2. pH, OM, the Activities of Urease and Phosphatase in Soil

Soil pH was measured with a pH meter (PHS-3C) using a 1:2.5 soil-to-water solution. Organic C was determined by dichromate digestion. Soil phosphatase activity was measured spectrophotometrically by the disodium phenyl phosphate method of Li [28]. Soil urease activity was determined using the indophenol blue colorimetric method [29]. Following collection, 100 mg of soil in a mixture of HF-HClO_4_-HNO_3_ was digested with a microwave digestion unit (Mars 5, CEM, USA). Specific steps were described in detail by Yuan *et al*. [30]. The clear digested samples were then transferred quantitatively to 25 mL calibrated flasks with deionized water. Blanks and a standard reference material were analyzed concurrently for accuracy assurance. A total of 18 composite soil samples were analyzed in triplicate. Concentrations of sixteen elements (Al, B, Ca, Cd, Co, Cr, Cu, Fe, K, Mg, Mn, Na, P, Pb, S, and Zn) in the soil samples were determined using ICP-OES (IRIS/AP, Thermo Jarrell Ash Corp., Franklin, MA, USA).

### 4.3. Analysis of the Bacterial Community Composition by PCR-DGGE

Total soil community DNA was extracted from 1.0 g soil using a bead beating method (UltraClean^™^ Soil DNA Isolation Kit, MoBio Laboratories, Inc., USA) following the manufacturer’s protocol. For bacteria DGGE analysis, the 16s rDNA was amplified with the primers F357GC-clamp and R518 [31].The structure of the dominant portion of the soil bacterial community was assessed using PCR-DGGE. Some prominent bands from the DGGE fingerprints were excised, cloned, sequenced and analyzed to get further information about the dominating bacterial populations of *F. thunbergii*. The details are described in Lin *et al*. [32].

### 4.4. Statistical Analysis

Data of element concentrations in soil samples were subjected to one-way analysis of variance (ANOVA) performed using SPSS 16.0 for Windows. In this study, pattern recognition methods including PCA have been applied. Samples were analyzed in triplicate and each analysis was inserted into the database as an individual data point.

## 5. Conclusions

Overall, there are obvious differences, not only between the rhizosphere and bulk soil but also among the regions. As a medicinal plant, *F. thunbergii* may secrete some unique metabolites in the root that exhibit anti-enzyme activity in rhizosphere soil. The soil properties and the plant species cooperatively shape the structure of the rhizosphere bacteria, among which proteobacteria, actinobacteria, acidobacteria and bacterioidetes were the main kinds of bacteria in the soil of *F. thunbergii*. Furthermore, the soil properties have a close relationship with the geoherbalism of *F. thunbergii*, especially pH, and we consider that alkaline pH may be more appropriate for the growth of *F. thunbergii*. Hence we draw a conclusion that the rhizosphere effect of *F. thunbergii* does exist but might be slightly different for a medicinal plant.

## Figures and Tables

**Table 1:** Concentrations of sixteen elements (mg kg^−1^) in rhizosphere and bulk soil of *F. thunbergii*, triplicate per sample, presented as means (standard error of mean; *n* = 3).

Element	Rhizosphere Soil in Nantong	Bulk Soil in Nantong	Rhizosphere Soil in Panan	Bulk Soil in Panan	Rhizosphere Soil in Ningbo	Bulk Soil in Ningbo
Al	5144 ± 402	3133 ± 376	8039 ± 366	3843 ± 249	9079 ± 427	5966 ± 310
B	3638 ± 262	2145 ± 285	1379 ± 404	570 ± 66	1215 ± 283	494 ± 76
Ca	2400 ± 166	2243 ± 225	1560 ± 71	994 ± 121	1763 ± 165	1031 ± 65
Fe	5041 ± 186	4856 ± 53	3637 ± 374	4734 ± 172	4705 ± 128	4349 ± 248
K	9261 ± 310	10,535 ± 458	13,393 ± 688	10,285 ± 321	11,450 ± 72	7139 ± 360
Na	3385 ± 348	4769 ± 169	5342 ± 470	4293 ± 252	5619 ± 188	3607 ± 323
Mg	309 ± 19	469 ± 16	75 ± 7	46 ± 11	168 ± 21	179 ± 15
Mn	258 ± 18	229 ± 5	167 ± 4	227 ± 30	199 ± 16	199 ± 17
P	546 ± 24	513 ± 13	369 ± 21	207 ± 11	915 ± 33	898 ± 24
S	162 ± 23	132 ± 7	91 ± 4	72 ± 4	183 ± 8	164 ± 2
Co	8.8 ± 0.5	10.2 ± 0.5	6.2 ± 0.6	7.3 ± 0.3	8.0 ± 0.7	9.2 ± 0.3
Zn	59.6 ± 7.9	71.1 ± 12.8	44.7 ± 10.5	38.4 ± 7.2	61.9 ± 4.9	55.1 ± 6.9
Cu	29.8 ± 2.5	36.4 ± 0.1	8.6 ± 0.2	3.9 ± 0.2	40.0 ± 1.3	17.6 ± 1.5
Cd	1.1 ± 0.1	0.5 ± 0.1	1.1 ± 0.0	0.7 ± 0.1	1.6 ± 0.1	0.8 ± 0.1
Cr	58.6 ± 6.1	57.7 ± 0.3	20.6 ± 0.9	22.1 ± 0.9	45.9 ± 3.2	34.4 ± 2.6
Pb	30.4 ± 2.8	26.8 ± 0.4	27.8 ± 2.1	23.8 ± 1.7	41.5 ± 3.9	30.9 ± 1.7

**Table 2:** The Pearson correlation analyses of soil properties in three regions of *F. thunbergii*.

	Ca	Fe	K	Mg	Mn	Na	P	S	B	Co	Zn	Cu	Cd	Cr	Pb	OM	pH	Phosphatase	Urease
Al	−0.26	−0.49*	0.36	−0.60**	−0.56**	0.41*	0.47*	0.27	−0.22	−0.60**	−0.19	−0.06	0.85**	−0.36	0.68**	0.53**	−0.73**	0.93**	0.24
Ca	1.00	0.47*	0.09	0.77**	0.51*	−0.14	0.00	0.42*	0.86**	0.46*	0.47*	0.72**	0.00	0.83**	0.13	−0.24	0.78**	−0.47*	0.63**
Fe		1.00	−0.44*	0.55**	0.84**	−0.43*	0.07	0.37	0.55**	0.59**	0.51*	0.57**	−0.15	0.75**	0.05	−0.23	0.63**	−0.62**	0.33
K			1.00	−0.20	−0.40	0.80**	−0.39	−0.44*	−0.08	−0.49*	−0.17	−0.07	0.20	−0.28	0.01	−0.15	−0.34	0.41*	−0.28
Mg				1.00	0.49*	−0.15	0.11	0.35	0.58**	0.86**	0.73**	0.77**	−0.48*	0.87**	−0.09	−0.14	0.90**	−0.67**	0.53**
Mn					1.00	−0.59**	−0.15	0.25	0.69**	0.49*	0.30	0.36	−0.20	0.69**	−0.15	−0.34	0.70**	−0.74**	0.23
Na						1.00	0.02	−0.24	−0.42*	−0.26	0.06	0.13	0.11	−0.26	0.18	0.05	−0.45*	0.57**	−0.16
P							1.00	0.84**	−0.11	0.31	0.36	0.53**	0.42*	0.29	0.74**	0.69**	−0.12	0.44*	0.73**
S								1.00	0.35	0.43*	0.42*	0.70**	0.43*	0.62**	0.70**	0.45*	0.25	0.11	0.89**
B									1.00	0.33	0.43*	0.50*	0.07	0.77**	0.01	−0.28	0.75**	−0.54**	0.56**
Co										1.00	0.70**	0.67**	−0.54**	0.76**	−0.06	0.03	0.75**	−0.61**	0.47*
Zn											1.00	0.78**	−0.20	0.74**	0.18	0.17	0.57**	−0.29	0.60**
Cu												1.00	0.08	0.88**	0.47*	0.13	0.55**	−0.17	0.81**
Cd													1.00	−0.13	0.74**	0.40	−0.51*	0.70**	0.37
Cr														1.00	0.17	−0.07	0.84**	−0.55**	0.74**
Pb															1.00	0.54**	−0.31	0.62**	0.63**
OM																1.00	−0.33	0.54**	0.37
pH																	1.00	−0.88**	0.41*
Phosphatase																		1.00	0.03

* Correlation is significant at the 0.05 level (2-tailed);

** Correlation is significant at the 0.01 level (2-tailed).

**Table 3:** Sequence alignment of 33 OTUs using Blast. Abbreviations: NT: The rhizosphere soil in Nantong; NT′: The bulk soil in Nantong; PA: The rhizosphere soil in Panan; PA′: The bulk soil in Panan; NB: The rhizosphere soil in Ningbo, NB′: The bulk soil in Ningbo. The position and brightness of bands on DGGE plots were digitized, 1 for when the band appeared and 0 for when none appeared.

Band	The Closest Sequences (GenBank Accession Number)	Length (bp)	NB	NB′	PA	PA′	NT	NT′
T1	Uncultured actinobacterium clone (EU715956)	170	0	0	1	0	1	1
			
T2	Uncultured Sphingomonadaceae bacterium clone (GQ338772)	169	0	0	1	1	1	0
			
T3	Uncultured Acidobacteria *bacterium* clone (EF663281)	193	0	0	1	1	1	1
			
T4	*Pseudomonas reactans* strain PSR2 (GQ354529)	194	0	0	1	1	1	0
			
T5	*Acinetobacter* sp. 71A1 (GQ178053)	195	0	1	1	1	1	1
			
T6	Uncultured Sphingobacteriales bacterium (FJ536881)	189	1	0	1	1	1	1
			
T7	Uncultured *Acinetobacter* sp. clone GI8-sp-H16 (GQ129954)	195	1	1	1	1	1	1
			
T8	*Flavobacterium columnare* strain QJH-2 (EU395803)		1	1	1	1	0	1
			
T9	*Acinetobacter* sp. 423D (GQ178046)	189	1	1	1	1	0	1
			
T10	Uncultured bacterium clone nbw691h12c1 (GQ116358)	195	0	1	0	0	0	0
			
T11	Uncultured soil bacterium C0165 (AF128666)	189	1	1	1	1	0	0
			
T12	Uncultured *Acinetobacter* sp. clone JEL30 (DQ130041)	194	0	0	1	0	0	0
			
T13	Uncultured Bacteroidetes bacterium (GQ469472)	195	1	1	1	1	1	1
			
T14	*Rhodanobacter* sp. IMER-B2-16 (FJ772029)	189	0	1	0	0	0	0
			
T15	Uncultured bacterium clone S5-92 (EU680352)	194	0	0	1	1	0	0
			
T16	Uncultured Rhizobiales bacterium clone P1s-1 (GQ287595)	169	0	0	1	0	0	0
			
T17	*Sphingomonas* sp. DX-T3-03 (GQ895737)	169	0	0	1	1	1	1
			
T18	Uncultured soil bacterium clone 20_5KE05 (GQ918621)	169	1	1	1	1	1	1
			
T19	Uncultured bacterium clone CafTC09 (GQ401700)	194	0	0	1	0	0	0
			
T20	Uncultured ammonia-oxidizing bacterium isolate (GQ281017)	194	0	0	0	1	1	1
			
T21	Uncultured proteobacterium clone (DQ223199)	194	1	0	0	0	1	0
			
T22	*Aquicella lusitana* strain SGT-39 (NR_025763)	193	0	0	1	1	0	0
			
T23	Uncultured Firmicutes bacterium clone (EU299094)	193	0	0	1	1	1	1
			
T24	*Micrococcus* sp. OS5 clone F12 (GU003860)	193	1	1	1	1	1	0
			
T25	Uncultured soil bacterium clone 21_77KB12 (GQ918955)	174	0	0	1	1	1	1
			
T26	Uncultured actinobacterium clone B10-05C (FJ543076)	170	0	0	1	1	1	0
			
T27	Uncultured bacterium clone C007 (GQ495796)	169	0	0	0	0	1	0
			
T28	Uncultured bacterium clone MACA-CC01 (GQ500694)	170	1	0	1	1	0	0
			
T29	Uncultured Acidobacteria bacterium clone (GQ288459)	194	1	1	1	1	0	0
			
T30	Uncultured soil bacterium clone 20_5KB11 (GQ918592)	169	0	1	1	1	0	0
			
T31	Uncultured actinobacterium clone H140 (GQ504251)	169	0	0	1	1	0	0
			
T32	Uncultured bacterium clone D10H_H03 (GQ376837)	174	1	1	1	1	1	1
			
T33	Uncultured Caulobacterales bacterium clone (EU665092)	169	0	0	0	0	1	0
